# Study of Adult and Pediatric Spanish Patients with Cryptogenic Splenomegaly and Splenectomy

**DOI:** 10.3390/diseases13040102

**Published:** 2025-03-30

**Authors:** Marta Morado Arias, Jesús Villarrubia Espinosa, Isidro Vitoria Miñana, Enrique Calderón Sandubete, Víctor Quintero, Miguel Ángel Torralba-Cabeza

**Affiliations:** 1 Department of Hematology and Hemotherapy, Hospital Universitario La Paz, 28046 Madrid, Spain; marta.morado@gmail.com; 2 Department of Hematology and Hemotherapy, Hospital Universitario Ramón y Cajal, 28034 Madrid, Spain; jesus.villarrubia@salud.madrid.org; 3 Nutrition and Metabolic Disease Unit, Hospital Universitario La Fe, 46026 Valencia, Spain; isivitoria@gmail.com; 4 Department of Internal Medicine and Biomedicine Institute, Hospital Universitario Virgen del Rocío, 41013 Seville, Spain; ecalderon@us.es; 5 Consejo Superior de Investigaciones Científicas (CSIC), Centro de Investigación Biomédica en Red Epidemiológica y Salud Pública de España (CIBERESP), 41013 Sevilla, Spain; 6 Department of Pediatric Hematology-Oncology, Hospital Universitario La Paz, 28046 Madrid, Spain; victormanuel.quintero@salud.madrid.org; 7 Department of Internal Medicine, Hospital Universitario Lozano Blesa, 50009 Zaragoza, Spain

**Keywords:** Gaucher, ASMD, splenomegaly, splenectomy, DBS

## Abstract

Introduction: The differential diagnosis of splenomegaly is a complex process that encompasses a wide variety of diseases. Moreover, it is not always standardized and lacks a definitive consensus on which tests should be performed and in what order. Gaucher disease (GD) and acid sphingomyelinase deficiency (ASMD) are lysosomal diseases (LD) that present with splenomegaly, the diagnosis of which requires a high index of suspicion and specific biochemical and genetic techniques. The aim of the project for the education and diagnosis of Gaucher disease and acid sphingomyelinase deficiency (PREDIGA) was to conduct educational training alongside an observational, multicenter, ambispective, cross-sectional, single-cohort study among patients having an enlarged spleen or undergone splenectomy to further assess these subjects to exclude two lysosomal diseases, namely GD and ASMD. Methods: Using dried blood spot (DBS) testing, we identified patients with abnormally low values of the enzymes glucocerebrosidase and acid sphingomyelinase, who then underwent sequencing of the GBA1 and SPMD1 genes, respectively. The study involved 34 hospitals and 52 medical specialists. Results: We identified 220 patients (208 adults and 12 children under 18 years) with cryptogenic splenomegaly or who had undergone splenectomy (12 patients) without having reached a diagnosis. The median age was 11 years (interquartile range [IQR] 3–16) in the pediatric population and 51 years (IQR 38–65) in the adult population. Lower-than-normal enzyme values were detected in 19 DBSs, confirming eight positive cases, which corresponded to six patients with GD and two with ASMD. The rest of the DBSs with low enzyme activity were not genetically confirmed (58%). We determined that lysosomal diseases accounted for 3.6% of cryptogenic splenomegaly/splenectomy cases in our setting: 2.7% were GD and 0.9% ASMD, in a ratio of 1 ASMD patient to every 3 GD patients. Lyso-GL1 values in patients with GD were elevated in all but one individual, corresponding to a child diagnosed at 4 months old. The variants detected in the GBA1 gene were consistent with the most frequent variants found in Spain. Discussion/Conclusion: The development and implementation of this protocol for the education and diagnosis of cryptogenic splenomegaly/splenectomy, even in asymptomatic patients, constitutes a comprehensive, simple, rapid, and effective screening method for the diagnosis of GD and ASMD.

## 1. Introduction

Splenomegaly is a very common clinical finding that may be present in up to 2% of the world’s population. Its etiology is highly variable and depends on the geographical area in which it occurs, with so-called tropical splenomegaly (malaria) being by far the most common cause in Africa and Asia. In Western countries, the most frequent causes are hematological, hepatic and infectious diseases, congestive or inflammatory causes and, in a small percentage of patients, metabolic diseases. In the vast majority of cases, splenomegaly is due to a systemic disease [1].

The definition of splenomegaly is unclear, although a spleen larger than 12–14 cm measured on its craniocaudal axis is usually accepted in adults, generally palpable ≥1 cm below the costal margin. It is palpable in between 5 and 10% of healthy children and in up to 30% of infants. The size of the spleen in children depends on age and body proportion, and there are several formulas for its calculation. The best way to measure splenomegaly is with abdominal ultrasound [2].

In adults, a palpable spleen is not synonymous with disease, since up to 16% of these organs are normal on ultrasound. Furthermore, in up to 28% of splenomegaly cases diagnosed by imaging techniques, the physical examination had been considered normal [3].

When examining a patient with splenomegaly, we must take into account the clinical context in which it occurs, so the differential diagnosis will depend on whether it presents together with fever, cytopenia, leukocytosis, thrombocytosis, lymphadenopathy, mediastinal masses, syndromic phenotypes, portal hypertension, etc., or in isolation.

The study of splenomegaly should be carried out in a stepwise manner depending on the form of presentation and should include the most basic studies (complete blood count, biochemistry, reticulocytes, peripheral blood smears) to the most sophisticated techniques, such as echocardiograms, bone marrow biopsy, or molecular techniques, among others [4].

Even so, between 10 and 20% of cases remain undiagnosed, a figure that has remained almost constant over the years [5]. This group includes lysosomal diseases, particularly Gaucher disease (GD) and acid sphingomyelinase deficiency (ASMD, formerly Niemann-Pick A, A/B or B disease), in which splenomegaly is the first sign of presentation in up to 80–90% of cases. Because they are diseases with very low prevalence and are therefore largely unknown, they are rarely considered [6].

With this background, and because rare diseases may be diagnosed in adulthood and the late-onset cases are often attenuated with very atypical presentation, we have proposed PREDIGA (project for the education and diagnosis of Gaucher disease and acid sphingomyelinase deficiency), an educational training associated with an investigative project. The two main objectives were

- To carry out educational training in centers of the Spanish public health network where there are doctors of different specialties with patients who present unaffiliated splenomegaly;
- To investigate if any of these patients could be affected by the GD and/or ASMD, by using enzymatic quantification with DBS testing as a diagnostic tool. Furthermore, we want to prove the usefulness of DBS testing as a simple, fast, and effective method for diagnosing and/or ruling out these two diseases. Analysis of the allelic variants found in confirmed GD or ASMD patients was performed.

## 2. Materials and Methods

### 2.1. Study Design

PREDIGA is an educational training parallel to an investigative, multicenter, ambispective, cross-sectional, single-cohort project. It involved 34 hospitals and 52 specialists, of whom 22 were hematologists, 18 internal medicine specialists, and 12 pediatricians.

The study was conducted in accordance with the requirements of the Declaration of Helsinki (Fortaleza-Brazil review, October 2013) for medical research involving human subjects. Likewise, the study was approved by the Ethical Committee of Hospital Ramon y Cajal (Madrid, Spain, 3 September 2019). All participants signed the informed consent before undergoing any procedure.

### 2.2. Medical Educational Part

For the educational part, a clinical training session was developed to raise awareness and knowledge of these 2 diseases, their diagnostic algorithms, and the different differential diagnoses of splenomegaly in pediatric and adult populations. These sessions lasted approximately 2 h and were given by investigators from the PREDIGA project. This training activity allowed the attendees to retrospectively identify potential patients from their clinical histories as well as new potential affected patients detected prospectively.

### 2.3. Investigative Part

#### 2.3.1. Inclusion and Exclusion Criteria

We included patients of any age with splenomegaly, defined as a palpable spleen ≥ 1 cm from the costal margin or above the normal limit for age as measured by imaging techniques, regardless of platelet counts. Subjects who had undergone splenectomy in whom a definitive diagnosis had not been obtained were also included. Patients with portal hypertension due to liver disease, hematologic neoplasms, or diagnosed with some type of congenital hemolytic anemia prior to study inclusion were excluded. Patients were selected by the physicians both retrospectively, from previous patients who were persistently undiagnosed, and prospectively, from new cases of patients with unrelated splenomegaly detected throughout the inclusion period of the study.

#### 2.3.2. Procedures

The number of hospitals participating was 34. Of these, 33 hospitals carried out a retrospective search for patients with the inclusion criteria and, in addition, the 34 participating hospitals initiated a prospective search for patients for 24 months.

The geographical distribution of the participating hospitals throughout Spain is shown in Supplementary Materials.

For the investigative part, each participating hospital identified patients with cryptogenic splenomegaly or splenectomy, that is, those patients in whom, after exhaustive investigation, the cause remained undetermined or not histologically confirmed. To that end, the medical records of each center were reviewed to identify retrospective cases, in addition to a 24-month prospective study of all new cases.

**Figure 1:**
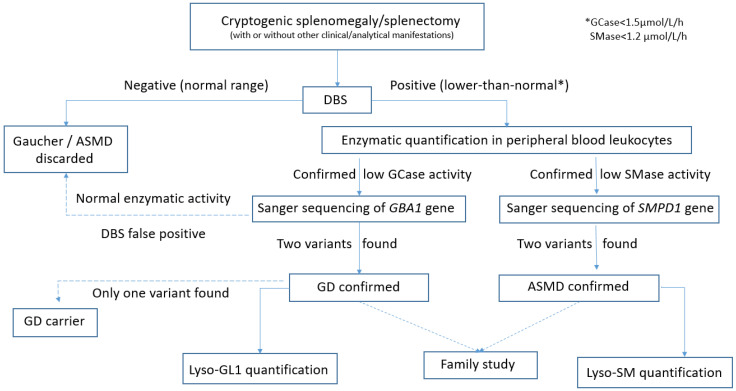
Flow diagram of the test and investigation procedures.

The study was conducted in a single patient visit. After signing the informed consent, clinical data were recorded, and peripheral blood was drawn for sample collection. The epidemiological parameters collected were age, sex, date of birth, date of splenomegaly diagnosis or splenectomy, as well as the definitive diagnosis obtained. Being an epidemiological trial, we focused on uncovering the incidence of two LDs among patients with cryptogenic splenomegaly or splenectomy, regardless of clinical manifestations. Hence, no additional clinical data were gathered in the data sheet, besides splenomegaly/splenectomy, either at the diagnosis or in the follow-up of those patients eventually diagnosed with GD or ASMD. The blood samples, freshly drawn and fixed on paper (dried blood spot, DBS), were processed in an accredited international laboratory (ARCHIMEDlife Laboratories^®^, Vienna, Austria) to simultaneously determine glucocerebrosidase (GCase) deficiency in the case of GD and acid sphingomyelinase (SMase) deficiency for ASMD using LC-MS/MS technology. GCase or SMase deficiency was defined as activity values below the low limit of normality, that is, less than 1.5 μmol/L/h and 1.2 μmol/L/h, respectively. If DBS results were positive or equivocal (enzyme activity less than or equal to the limit of normal), new samples were reanalyzed at the same international laboratory to quantify the activity of the enzymes GCase and acid SMase in peripheral blood leukocytes. Sequencing by Sanger of the GBA1 and SMPD1 genes was performed for those patients with confirmed enzymatic deficiency, using the same sample. GD and ASMD diagnoses were confirmed when two pathological variants were detected in these genes. Carrier status was made if only one variant was detected. In the positive cases, the same laboratory provided the baseline lyso-GL1 (GD) and lyso-SM (ASMD) values for subsequent monitoring. To avoid errors in the genetic diagnosis, all the pathogenic variants detected were investigated in the parents of the patients, except in those who had already died. A flow chart of the study procedure is shown in Figure 1.

#### 2.3.3. Statistical Analysis

We used the statistical package SPSS version 24. Continuous variables were described by mean and standard deviation (SD) in the case of normal distribution or by median and interquartile range (IQR) if data were not normally distributed. Categorical variables were analyzed by frequency and percentage.

## 3. Results

The study was conducted from September 2019 to March 2022, obtaining the following results.

### 3.1. Medical Educational Part

Fifty clinical sessions were held in 42 hospitals with around 1000 physicians in attendance. This training activity allowed one of the attendees to recognize a suspected GD or ASMD and to send the sample to be analyzed for confirmation.

### 3.2. Investigative Part

We identified 220 patients with cryptogenic splenomegaly or splenectomy (208 adults and 12 children under the age of 18). Twelve adults had undergone splenectomy, while the remainder corresponded to patients with splenomegaly with no etiological diagnosis (196 adults and 12 children). The median age was 11 years (IQR 3–16) for the pediatric population (seven girls/five boys) and 51 years (IQR 38–65) for the adult population (153 men/55 women). The median age at splenomegaly diagnosis was 10 years (IQR 1–15) in the pediatric population and 48 years in adults (IQR 34–63). Splenectomies were performed at age 37 (IQR 28–48), two of them when the patients were 13 and 15 years old. None of the pediatric patients had undergone splenectomy. Demographic details are shown in Table 1.

Of the 220 patients studied, lower-than-normal enzyme values were detected in 19 DBSs. After enzymatic and genetic analysis, eight positive cases were confirmed, corresponding to six patients with GD and two with ASMD, all diagnosed prospectively. Considering the cases analyzed, lysosomal diseases accounted for 3.6% of cryptogenic splenomegaly/splenectomy patients in our population, with 2.7% corresponding to GD and 0.9% to ASMD. The ratio of diagnoses was 1 case of ASMD to 3 cases of GD. The participating hospitals covered a total of 14,174,510 inhabitants, so the incidence of new diagnoses of GD and ASMD within cryptogenic splenomegaly/splenectomy in our population was 0.4 cases and 0.14 cases per million inhabitants, respectively.

When analyzing the eight patients diagnosed with GD or ASMD, it was shown that all had splenomegaly, but none had undergone splenectomy. These cases were detected by two pediatricians, two hematologists, and four internal medicine specialists participating in the study. Those patients with the longest duration of unspecified splenomegaly were easily identified retrospectively: a 60-year-old woman with ASMD and a 71-year-old male with GD. The remaining cases were identified prospectively. Specific details regarding clinical date, age of diagnosis, reason for screening, and biochemical and genetic results of these eight patients are shown in Table 2.

The age at which the diagnosis was made was highly variable: four patients were diagnosed in the year after the splenomegaly was diagnosed, when they were immediately enrolled in the study; another three were diagnosed in the 5 years following the detection of splenomegaly; and at the extreme end, we had the case of another patient in whom it took 39 years to be diagnosed with ASMD (Table 2).

Quantification of enzyme activity showed a mean glucocerebrosidase value of 0.35 µmol/mL/h (normal ≥ 1.5) in patients with GD and a mean sphingomyelinase value of 0.45 µmol/mL/h (normal ≥ 1.2) in ASMD, clearly lower than in other cases without lysosomal disease (Table 2).

Lyso-GL1 values in patients with GD were abnormal in all but one case, corresponding to a child diagnosed at 4 months old. The remaining patients had lyso-GL1 values above normal (normal < 14 ng/mL), with a mean of 457.40 ng/mL (range: 17.6–851.80) (Table 2). Lyso-SM was above the normal range (normal < 70 ng/mL) in one of the ASMD patients.

The study identified another 11 cases in which the DBS had below-normal quantification of enzyme activity without genetic confirmation of GD or ASMD (10 DBS with glucocerebrosidase < 1.5 µmol/mL/h; 1 DBS with sphingomyelinase < 1.2 µmol/mL/h). This accounts for 5% of the samples analyzed. Only 42% of patients with a lower-than-normal DBSs (8/19) were diagnosed with lysosomal disease, resulting in 58% abnormal DBSs without molecular confirmation. It should be noted that among those with a low glucocerebrosidase value, a carrier of the c.1226A>G variant of the GBA1 gene was identified, with no second variant being detected so far.

The genetic variants of patients with GD and ASMD are listed in Table 2. In patients with GD, the most frequently detected GBA1 gene variant was c.1226A>G (formerly known as N370S) in 66% (4/6) of patients (5/12—42% of alleles), followed by c.1265_1319del55 and c.1448T>C (formerly L444P), both detected in two patients (33%). With regard to variants in the SMPD1 gene, notably, homozygosity for c.1744C>A was identified in one patient, with the other two variants being deletions in the second patient.

Conclusions cannot be drawn regarding the definitive diagnoses of patients in whom GD or ASMD were not confirmed using the current data, since it was not the aim of the study. Information was obtained on the suspected diagnosis of splenomegaly in 40 patients (40/212; 19%). The main suspected or confirmed causes were: chronic liver disease (nine cases: idiopathic, alcoholic, cardiac), hematologic neoplasms (eight cases: lymphomas, myeloproliferative or myelodysplastic syndromes), infections/inflammation (five cases: Q fever, Pontiac fever, cytomegalovirus, HIV), congenital hemolytic anemia (four cases), autoimmune disease or cytopenia (five cases), congenital deficiencies of other enzymes (three cases: one lecithin-cholesterol acyltransferase deficiency, one alpha-1 antitrypsin deficiency, and one tuberous sclerosis), cysts (two cases), and non-hematologic neoplasms (one hemangioblastoma). Seventy-eight percent of splenomegaly or splenectomized patients (172/220) remain without a confirmed or probable diagnosis, some of them for more than 30 years.

## 4. Discussion

The differential diagnosis of pathological enlargement of the spleen encompasses a wide variety of diseases [5]. It is a complex process, not always standardized, without a definitive consensus on which tests and in what order they should be performed [7]. From a clinical perspective, there are specific diagnostic methods in the case of pediatric patients who have splenomegaly with fever, lymphadenopathy, mediastinum masses, or a particular phenotype. However, there are two subgroups of patients with splenomegaly or splenectomy in which the diagnostic challenge is greater: (1) those with cytopenia and (2) those with isolated splenomegaly [4,8]. These subgroups included patients with lysosomal diseases, in which a delay in diagnosis of GD and ASMD complicates treatment, causes greater organic damage, or even leads to unnecessary splenectomy. The lysosomal diseases ASMD and GD are both macrophage storage disorders with overlapping clinical manifestations, such as bone complications, hepatosplenomegaly, thrombocytopenia, anemia, and pulmonary involvement [9,10]. Therefore, differential diagnosis should be part of common clinical practice among patients with splenomegaly and/or some of these clinical features [11].

In our study, we have carried out educational work to raise awareness among physicians of the importance of screening lysosomal diseases in patients with cryptic splenomegaly, a strategy that is innovative and not found in the literature. After this educational work, in the investigative part, we have shown that testing retrospectively as well as prospectively for glucocerebrosidase and acid sphingomyelinase activity using DBS testing should become a routine practice to characterize splenomegaly of unknown etiology. In important to highlight that our study recruited patients with cryptogenic splenomegaly regardless of the presence of additional symptoms such as thrombocytopenia, which allows us to identify patients who would not be detected if other clinical criteria were included. Based on a sample of 220 patients with cryptic splenomegaly/splenectomy, a definitive diagnosis was reached in eight subjects, six of them with GD (four patients without thrombocytopenia) and two with ASMD. This means that lysosomal diseases were identified in 3.6% of patients with cryptogenic splenomegaly or splenectomy, the most common being GD (2.7% of splenomegaly/splenectomy cases). The incidence of ASMD was much higher than expected (0.9% of studies) compared to GD, with about one case of ASMD being identified for every three GD [10]. It has taken decades for some of these patients to be diagnosed, which adds value to these screening measures, not only in children but also in adults.

Our results are concordant with those published recently in the literature, even though many of them only cover pediatric patients. Young Rok Do et al. [12] performed a multicenter, observational study conducted at 18 sites in Korea among adult patients with unexplained splenomegaly who were enrolled and tested for B-glucosidase enzyme activity in DBSs. A total of 352 patients were enrolled, but only one female was diagnosed with type 1 GD, and no screening for ASMD was performed. Unlike our work, it showed much less effectiveness. In Di Rocco’s study [13], visceromegaly in the pediatric population was chosen as the starting point of the algorithm, being the most frequent sign of onset for both GD and ASMD. Then, the authors identified two parallel diagnostic pathways for GD (assessed by the presence of Erlenmeyer flask deformity or growth retardation or increased ferritin concentration) or ASMD (assessed by the presence of Interstitial lung disease or growth retardation or hypoalphalipoproteinemia) which finally concluded with the completion of DBS testing. Despite being an acceptable protocol, we do not consider the possibility of parallel diagnoses, and we directly screen for both entities using DBSs, based only on the presence of splenomegaly, regardless of other clinical manifestations, to increase the diagnostic yield and simplify the screening method. In the study conducted by Pession et al. [14], whose aim was the early diagnosis of GD in children with unexplained splenomegaly and thrombocytopenia, they collected DBS samples and tested for β-glucocerebrosidase enzyme activity in 154 patients selected through the algorithm proposed by Di Rocco et al. [13]. Although the study was shown to be effective, the authors acknowledged that the protocol was designed for use by hematologic pediatricians in children with splenomegaly and thrombocytopenia, with limitations regarding its use in adults. In 2024, Giacomarra et al. [15] studied a total of 627 samples of patients with an initial clinical suspicion of GD from hematology, internal medicine, and pediatric centers throughout Italy, using a DBS test. A total of 8 of the 627 patients (1.3%) had deficient glucocerebrosidase activity, which confirmed the initial clinical suspicion of GD, and in three subjects (0.5%), a reduction in acid sphingomyelinase activity was detected, a lower rate than in our study.

As demonstrated in the literature and corroborated in our study, the DBS technique is a standardized, non-invasive, and low-cost diagnostic screening method [16]. However, as demonstrated in our study, given the high false lower-than-normal rate (no genetic alteration was found in 58% of the DBS quantifications below the limit of normal), it is essential to confirm in fresh blood leukocytes all cases that show low activity on DBS testing and/or with molecular genotyping. Similar results were detected in similar studies, in which the low DBS rate, no confirmed genetically rise as high as 80% even in patients with associated thrombocytopenia [17].

Regarding GD, the most frequently detected variants corresponded to the c.1226A>G allele, which was identified in five patients with GD type 1 and the mild phenotype. This was followed by a 55-base pair deletion in exon 9 of the GBA1 gene, which is particularly common in the Spanish population, and was found in two of our patients [18]. This deletion gives rise to a truncated protein and, in the case of one of the carriers, was detected as part of a phenotype compatible with GD type 3; the patient presented another recombinant allele without the residual protein that involved the c.1448T>C variant (previously designated L444P). The other patient with the aforementioned 55-base pair deletion had a mild phenotype as a result of mutation c.1604G>A in the second allele; it is considered mild since it produces a change from an Arginine to a Histidine at codon 535 of the GBA protein (https://clinvarminer.genetics.utah.edu, accessed on 24 March 2025). Other alleles detected were recombinant gene/pseudogene allele RecC5a, isolated mutation c.1448T>C (formerly L444P), and c.706C>T, which is considered mild due to minimal evidence of alterations in protein function when a Leucine amino acid is replaced by a Phenylalanine at position 236 of the polypeptide chain (https://clinvarminer.genetics.utah.edu, accessed on 24 March 2025). Regarding the SMPD1 gene, we detected (1) the pathogenic variant c.1547A>G, which replaces a Histidine with an Arginine at codon 516. This creates or strengthens a splice site, disrupting protein function; (2) a null allele caused by a deletion of the gene; and (3) another variant, c.1744C>A, which creates a Proline-to-Threonine change previously considered a variant of uncertain significance due to minimal structural, functional, and spatial involvement from the point of view of predictive studies (https://clinvarminer.genetics.utah.edu, accessed on 24 March 2025).

In terms of limitations, it is essential to be able to extend this project—both educational and screening aspects—to all hospitals in the Spanish health network, since it has been limited to the referral hospitals that were selected in the initial design. Especially important from the point of view of the project is the prompt diagnosis at an early age of ASMD, the treatment of which would prevent pulmonary and lipid complications. In the case of GD, its early treatment allows better bone growth and mineralization together with normalization of visceral, growth, and hematological alterations [19]. Another set of limitations lies in the interpretation of diagnostic tests, which requires highly qualified, specialized, and trained personnel. There is also another barrier, which is the large amount of time that must be dedicated both to the training sessions and to each patient, not because of the time it takes to obtain the test results, but because, apart from the study of enzyme activity, it is not clear what other tools to use and what other diagnostic techniques to carry out in patients who remain undiagnosed. Finally, we are aware that the population sample used in our project is small, and that the final objective must be to continue with the diagnostic process to evaluate the clinical utility and diagnostic yield of each of the tests carried out. We also recognize that it would have been interesting to have clinical and laboratory data at diagnosis and follow-up of the patients with lysosomal diseases, which is a limitation of this study. Despite the early incorporation of tests aimed at ruling out GD and ASMD in the study of splenomegaly, a significant number of patients still do not have a definitive diagnosis, confirming the diagnostic difficulty in these patients. Initiatives similar to the PREDIGA project in the form of a simplified screening for other rare diseases could help solve this clinical problem.

Our work can be easily reproduced and used by both primary care physicians and specialists in pediatrics, hematology, and internal medicine in different health institutions thanks to the fact that the study is based on collecting and sending DBS samples. In this regard, the implementation in the near future of a similar educational and research project has been planned in other Spanish hospitals that have been unable to participate to date, with a special emphasis on the educational aspects of doctors in training.

## 5. Conclusions

Our results reflect that GD and ASMD represent a significant percentage of the causes of unexplained splenomegaly, both in adults and in children, regardless of the co-existence of additional clinical findings. For their correct identification, it is essential to train physicians about the need to screen for these two entities among cases of cryptic splenomegaly, to avoid diagnostic splenectomy. Enzymatic quantification of GCase and SMase using DBS is a rapid, sensitive, effective, and simple technique that should be routinely used in the diagnosis of these patients.

## Figures and Tables

**Table 1:** Demographic data of the 220 patients included in the PREDIGA study.

	Total Cohort of Patients N = 220	Patients Without Diagnosis N = 212	Patients Diagnosed GD/ASMD N = 8
Gender			
Male n (%)	158 (72)	154 (73)	4 (50)
Female n (%)	62 (28)	58 (27)	4 (50)
Age at study inclusion			
Adults n (%)	208 (95)	202 (95)	6 (75)
Children n(%)	12 (5)	10	2 (25)
Reason for screening:			
-Crytogenic splenomegaly n (%)	208 (95)	200 (94)	8 (100)
Adults n (%)	196 (94)	190 (95)	6 (75)
Children n (%)	12 (6)	10 (5)	2 (25)
-Splenectomized n (%)	12 (5)	12 (6)	0
Adults n (%)	12 (100)	12 (100)	0
Children n (%)	0 (0)	0 (0)	0

**Table 2:** Data of patients diagnosed with ASMD or GD.

Diagnosis	Clinical Features	GCase μmol/L/h (N ≥ 1.5)	SMase umol/L/h (N ≥ 1.2)	LGL-1 ng/mL (N < 14)	L-SM ng/mL (N < 70)	***GBA1*** Gene Variants	***SMPD1*** Gene Variants
**ASMD**	60 y-o. female Splenomegaly diagnosis at age 22 y-o ASMD diagnosis after 39 years of cryptogenic splenomegaly		0.3		327		c.1829_1831delGCC; p.Arg610del c.1547A>G; p.His516Arg
**ASMD**	69 y-o male Splenomegaly diagnosis at 68 y-o ASMD diagnosis after 3months of cryptogenic splenomegaly		0.6		41		c.1744C>A; p.Pro582Thr c.1744C>A; p.Pro582Thr
**GD III**	4 month old male Splenomegaly diagnosis at 2 m-o Initially suspected pancytopenia secondary to CMV infection GD type III diagnosis after 2 months	0.9		2.4		c.1265_1319del55; p.Leu422Pro*fs c.(1448T>C; 1483G>C; 1497G>C); p.(Leu483Pro; Ala495Pro; Val499=)	
**GD I**	53 y-o female Splenomegaly diagnosis at age 53 y Associated with pancytopenia GD type I diagnosis after 2 months	0.0		851.8		c.1226A>G; p.Asn409Ser c.706C>T; p.Leu236Phe	
**GD I**	14 y-o female Splenomegaly diagnosis at 13 y-o Associated with thrombocytopenia Bone marrow morphology: Gaucher cells GD type I diagnosis after 14 months	0.0		839.7		c.1226A>G; p.Asn409Ser c.(475C>T+667T>C+681T>G+689T>G+703T>C+721G>A+754T>A); p.(Arg159Trp+Trp223Arg+Asn227Lys+Val230Gly+Ser235Pro+Gly241 Arg+Phe252lle)	
**GD I**	71 y-o. male Splenomegaly diagnosis at 66 y-o GD type I diagnosis after 5 years of cryptogenic splenomegaly	0.0		229.9		c.1226A>G; p.Asn409Ser c.1226A>G; p.Asn409Ser	
**GD I**	65 y-o male Splenomegaly diagnosis at 64 y-o Associated with thrombocytopenia GD type I diagnosis after 2 years	0.4		17.60		c.1226A>G; p.Asn409Ser c.1448T>C; p.Leu483Pro	
**GD I**	20 y-o female Splenomegaly diagnosis at age 20 y Associated with pancytopenia GD type I diagnosis after 1 month	0.8		348.0		c.1265_1319del55; p.Leu422Pro*fs c.1604G>A p.Arg535His	

## Data Availability

The datasets used and analyzed during the current study are not publicly available to preserve individuals’ privacy under the European General Data Protection Regulation. The datasets are available from the corresponding author on reasonable request.

## Supplementary Materials

The following supporting information can be downloaded at: https://www.mdpi.com/article/10.3390/diseases13040102/s1, Figure S1: Geographical distribution of the participating hospitals.

## Abbreviations

The following abbreviations are used in this manuscript:

GD	Gaucher disease
ASMD	Acid sphingomyelinase deficiency
LD	Lysosomal diseases
DBS	Dried blood spot
GCase	Glucocerebrosidase
SMase	Sphingomyelinase
SD	Standard deviation
IQR	Interquartile range
