# Supplementary figures and images for: Study of Adult and Pediatric Spanish Patients with Cryptogenic Splenomegaly and Splenectomy
Source: Diseases. 2025 Mar 30;13(4):102. doi: 10.3390/diseases13040102 (PMC12025833; doi:10.3390/diseases13040102)

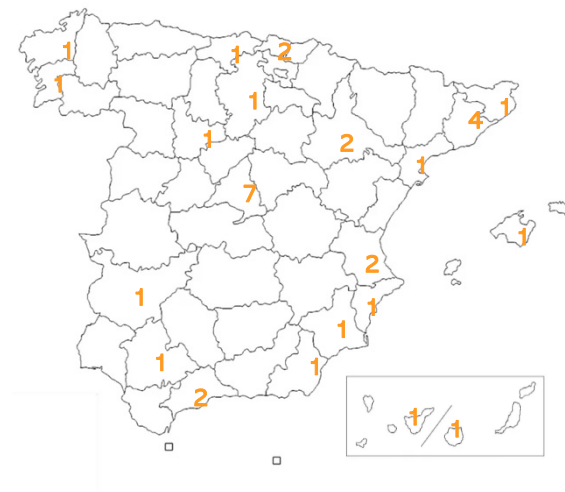

**SUPPLEMENTARY Figure S1. Geographical distribution of the participating hospitals**

Supplement: Supplementary file 1 [file diseases-13-00102-s001.zip › diseases-3505738-supplementary.pdf]
